# 807 Profound Role of a Survivor Adaptive Sports Program on Quality of Life and Self Perception

**DOI:** 10.1093/jbcr/iraf019.338

**Published:** 2025-04-01

**Authors:** Roselle Crombie, Philip Fidler, Cindy Rutter, Josh Mishell, Thereasa Abrams

**Affiliations:** Connecticut Burn Center, Bridgeport Hospital / Yale-New Haven Health; REACH Burn Foundation; 1Amazing Life; Fermentable Sugar LLC; University of Tennessee, Knoxville

## Abstract

**Introduction:**

Mental health struggles amongst adult burn survivors has recently been documented as an ongoing need for therapy in peer reviewed journals. Although robust programs exist for children, there is a paucity of those for adults. Ten years ago, a subset of this author group ran an adult adaptive sports survivor program in conjunction with a national burn meeting. All the participants, mental health providers and participating surgeons experienced the profound impact a program can have on the mental outlook, return to vocation and improvement in their survivor’s lives. Participants could include amputees and larger TBSA individuals.

**Methods:**

Motivation to create a more sustaining survivor retreat was high. A non-for profit for adult survivors was created and program developed. Applicants included adult burn survivors, > 26yo, greater than one year from injury. Additionally, two certified mental health professionals were on site to participate in the therapy part of the programing, as well as volunteers who were long time burn survivors, a fireman, other Burn RNs with two practicing burn surgeons.

On site prior to starting the retreat, participants were surveyed on topics including expectations of the event, mental and physical status, perception of self, and support system. All participants were then surveyed immediately after the event, then 30 days post event. A well validated Quality of Life scale was utilized.

**Results:**

Ten culturally heterogeneous burn survivors participated, five men and five women ranging in age from 27 to 62-years-old. Deficits ranged up to 80% TBSA survivors with varying limitations in mobility. Burn survival years ranged from 1 year-40 years post burn injury. Post program survey revealed mental health benefits including camaraderie, confidence to do activities they never participated in before and increase in self-esteem. Participants noted the profound impact of identifying a community of support for their ongoing healing journey. Changed perception of self and self-value was experienced by each survivor.

**Conclusions:**

Because sustainable mental health programs is an unmet need for adult burn survivors, programs such as the REACH for Resilience retreat play an important role in this unique population. The immediate change in each survivor’s outlook was impactful. Ongoing follow-up surveillance regarding the impact of this experience on quality of life will be done. The positive impact the event had on the volunteers and staff was unexpected and noteworthy. The authors are dedicated to refining the content of these retreats as therapeutic interventions in perpetuity given their effectiveness.

**Applicability of Research to Practice:**

Burn surgery has done a fantastic job of getting our larger burns to survive but the beginning of their actual healing journey starts with their discharge. Ongoing research on this topic can help to raise support for these long term programs and improve quality of life.

**Funding for the Study:**

N/A

